# Anticancer and Antimicrobial Activity of *Chlorella vulgaris* BA02 Algae Extract Containing Indole-3-Acetic Acid

**DOI:** 10.3390/molecules31020275

**Published:** 2026-01-13

**Authors:** Agata Jabłońska-Trypuć, Urszula Wydro, Elżbieta Wołejko, Paweł Kondzior, Maja Leszczyńska, Carmen Estevan Martínez, Özge Karakaş Metin, Marzena Ewa Smolewska, Rafał Krętowski, Marzanna Cechowska-Pasko, Adam Cudowski

**Affiliations:** 1 Department of Chemistry, Biology and Biotechnology, Faculty of Civil Engineering and Environmental Sciences, Bialystok University of Technology, Wiejska 45E Street, 15-351 Bialystok, Poland; u.wydro@pb.edu.pl (U.W.); e.wolejko@pb.edu.pl (E.W.); p.kondzior@pb.edu.pl (P.K.); 87023@student.pb.edu.pl (M.L.); 2 Departamento de Biología Aplicada, Instituto de Bioingeniería, Universidad Miguel Hernández de Elche, 03202 Elche, Spain; cestevan@umh.es; 3 Department of Molecular Biology and Genetics, Çanakkale Onsekiz Mart University, Çanakkale 17000, Turkey; ozgekarakasmetin@comu.edu.tr; 4 Department of Agri-Food Engineering and Environmental Management, Bialystok University of Technology, Wiejska 45E Street, 15-351 Bialystok, Poland; m.smolewska@pb.edu.pl; 5 Department of Pharmaceutical Biochemistry, Medical University of Bialystok, Mickiewicza 2A Street, 15-222 Bialystok, Poland; r.kretowski@umb.edu.pl (R.K.); marzanna.cechowska-pasko@umb.edu.pl (M.C.-P.); 6 Department of Water Ecology, Faculty of Biology, University of Bialystok, Ciolkowskiego 1J Street, 15-245 Bialystok, Poland; cudad@uwb.edu.pl

**Keywords:** algae, *Chlorella vulgaris*, IAA, bacteria, cancer, apoptosis, oxidative stress, cytotoxicity

## Abstract

In recent years, the incidence of breast cancer has increased significantly; therefore, much attention is being paid to research on natural plant-based raw materials in the treatment and prevention of cancer as well as in the treatment of antibiotic-resistant infections. Therefore, *Chlorella vulgaris* algae extract and indole-3-acetic acid (IAA)—a plant hormone with potential anticancer and antimicrobial properties—were selected for the study. The main objective was to evaluate the effect of algae extract and IAA on the proliferation of cells from three different breast cancer lines: MCF-7, ZR-75-1, and MDA-MB-231. In addition, an analysis of apoptosis and oxidative stress parameters in cancer cells was performed, as well as an assessment of IAA toxicity towards *E. coli*, *S. aureus*, and *C. albicans*. The results obtained allow us to conclude that the extract is effective against estrogen-dependent cells, while the effect of IAA alone varies depending on the microorganism studied, the cell line analyzed, and the concentration used. The extract in selected concentrations induces apoptosis and activates oxidative stress mechanisms, while IAA exhibits cytotoxicity at higher concentrations and stimulates proliferation at lower concentrations. This indicates the need to investigate the mechanisms of action of both *Chlorella vulgaris* algae extract and IAA in cancer and bacterial cells.

## 1. Introduction

Two serious problems that our world is facing now are cancer and antibiotic-resistant infections. The increase in the incidence of various types of cancer is a persistent health problem worldwide. Among women, breast cancer is the most common type of cancer, which results in very high mortality [1]. According to the data from the national cancer registry for 2017, in Poland, breast cancer accounted for 22% of the total number of cancer cases, and half of this number are fatalities. However, the reason for such a high incidence of this type of cancer has not been defined. Risk factors include age, genetic predisposition, long-term hormonal contraception, being overweight, use of hormone therapy, and leading an unhealthy lifestyle. By popularizing preventive breast examination and increasing awareness, cancer can be diagnosed at an early stage and appropriate treatment can be initiated, which gives a good prognosis for the patient. On the other hand, commonly used antibiotics are losing their ability to combat bacterial infections. Therefore, currently, many scientific studies are being conducted to identify chemotherapeutics that will constitute an effective anticancer therapy and antimicrobial therapy. The search for effective compounds with anticancer activity and low cytotoxicity towards healthy cells is a very important aspect of these analyses. Due to the high interest in a healthy diet rich in natural plant products and its potential preventive role in cancer, an extract from the algae species *Chlorella vulgaris* was tested. It contains, among other things, a plant-derived compound from the auxin group—plant hormones responsible for the proper growth and development of plants. This compound, indole-3-acetic acid (IAA), has been shown to have a potentially cytotoxic effect on cancer cells, although its effect is significantly less than that of the whole extract.

All species of algae are the main producers of organic matter in the aquatic environment, simultaneously serving as a primary source of food and oxygen for animals. Due to their very rich chemical composition, which includes easily digestible proteins, carbohydrates, lipids, vitamins, and mineral salts, they constitute a source of biologically active substances important from a dietary perspective. Consequently, their applications are very broad, encompassing food, pharmaceutical, cosmetic, and even animal feed production [2,3]. Among algae, many species have been identified as rich in chemically diverse organic compounds with a wide range of pharmacological activity. Some synthesize metabolites with cytostatic properties and anticancer compounds, as well as glycoproteins with anti-inflammatory and antibiotic properties, and those that activate immune processes. *Chlorella* is an example of such an alga [4,5,6].

**Figure 1:**
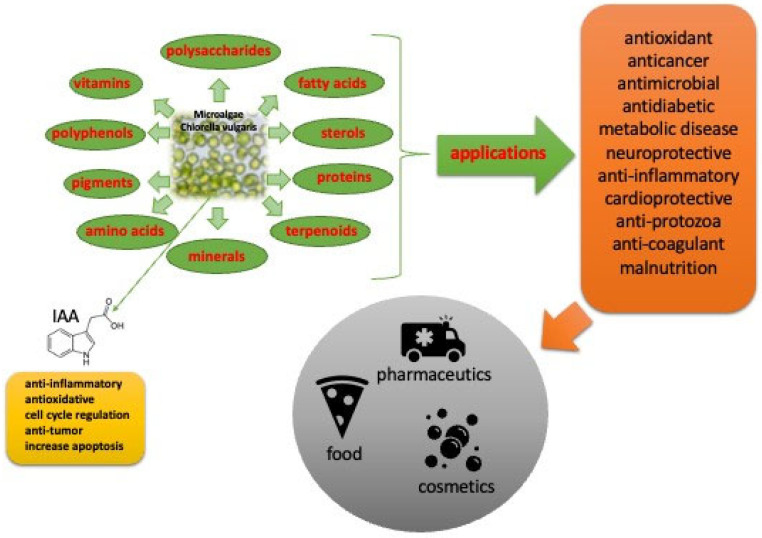
*Chlorella vulgaris* extract chemical composition and its biological activity with special attention paid to IAA as one active ingredient in the extract.

Algae, like higher plants, are also an important source of phytohormones. Plant hormones, also known as phytohormones, are the most important factors influencing plant growth and development. The most common auxin naturally occurring in algae/plants is indole-3-acetic acid (IAA). It can be produced not only by algae/plants but also by microorganisms such as *Bacillus* sp., *Pseudomonas* sp., *Enterobacter* sp., and *Rhisobium* sp., which promote plant growth (Figure 1) [7].

Indole-3-acetic acid, in addition to its positive effect on plant growth and development, also exhibits potential anticancer activity, but only after oxidation by horseradish peroxidase (HRP) [8]. IAA conjugated with horseradish peroxidase is a potential prodrug in aanticancertherapy.

Literature data indicate a significant effect of IAA on the inhibition of enzymes that have a key impact on the growth and metabolism of many microorganisms, causing metabolic imbalances and ultimately leading to their death. The group of microorganisms in which oxidative stress and membrane damage are observed after IAA application includes *Escherichia coli* and *Pseudomonas aeruginosa*, *Staphylococcus* spp., and *Streptococcus* spp. [9,10].

The main objective of the study was to evaluate the antibacterial and anticancer activity of *Chlorella vulgaris* algae extract and the in vitro toxicological activity profile of indole-3-acetic acid (IAA), as well as to check whether IAA is responsible for the bioactivity of algal extract. The research hypothesis and expected results were based on the assumption that the algae extract, rich in biologically active substances, including IAA, and IAA itself, would inhibit the proliferation of bacterial/fungal cells representing common pathogens and cells from the most common solid tumors currently occurring—breast cancer (estrogen-dependent and triple-negative), and induce cancer cell death. The intriguing question was whether the extract or the IAA compound itself would prove more effective. Therefore, the effect of *Chlorella vulgaris* extract and indole-3-acetic acid on the viability of bacterial/fungal cells and the proliferation of estrogen receptor-positive (ER+) and estrogen receptor-negative (ER−) breast cancer cells was investigated. This allowed for the preliminary determination of the activity of the analyzed extract and compound as a natural chemopreventive agent reducing the viability of bacterial/fungal and cancer cells.

## 2. Results

### 2.1. Chemical Analysis of Algae Extract

Qualitative GC/MS analysis has revealed and confirmed the very rich qualitative composition of *Chlorella vulgaris* extract (Table 1). Unsaturated fatty acids are quantitatively dominant and are certainly one of the most important groups of compounds that modulate human health. Among them, we can distinguish α-linolenic acid, oleic acid, and palmitoleic acid. There are also many sugars such as fructofuranose and glucopyranose. The extract also contains many amino acids, making it an excellent source of these essential building blocks for the human organism. Such a rich composition determines the highly effective action of the tested extract on cancer cells.

Quantitative LC/MS analysis identified IAA in the tested extract at a concentration of **1.3 μM (0.22 μg/mL)**.

### 2.2. Cytotoxicity Assays in Cancer Cells

**Figure 2:**
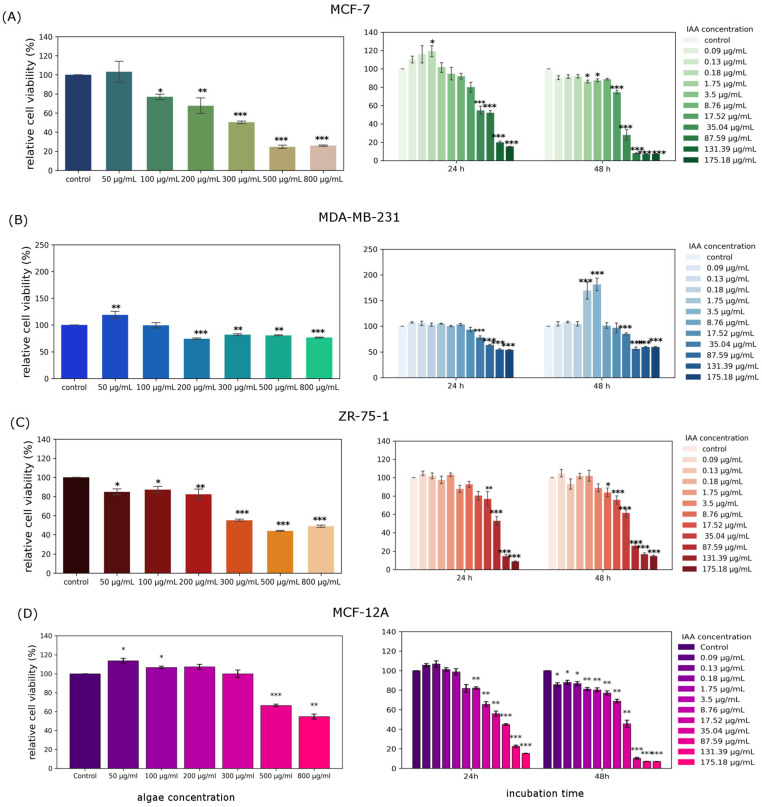
*Chlorella vulgaris* extract cytotoxicity and IAA cytotoxicity studied in MCF-7 (**A**), MDA-MB-231 (**B**), ZR-75-1 (**C**), and MCF-12A (**D**) cell lines, calculated as percentage of control cells. Data are presented as mean ± standard error of mean of four replicates. *—*p* < 0.05, **—*p* < 0.01, ***—*p* < 0.001 represent statistically significant differences from control, assessed by Dunnett’s test.

The cytotoxicity of the analyzed extracts was tested using the MTT assay (Figure 2). The incubation time was 24 h for each cell line. In the MCF-7 cell line, the IC50 concentration was achieved at 300 µg/mL, similar to the ZR-75-1 cell line. In the MCF-7 cell line, only one increase in cancer cell viability compared to the control was observed after exposure to the tested extract at the concentration of 50 µg/mL, and was statistically insignificant. In the ZR-75-1 cell line, no increase in cancer cell viability was observed following treatment with the *Chlorella vulgaris* extract. The IC50 was also 300 µg/mL. In the case of the MDA-MB-231 line, the greatest decrease in viability, approximately 25%, was the result of cell treatment with *Chlorella vulgaris* extract at a concentration of 200 µg/mL. However, in the case of the MCF-12A line, which is a healthy line, a significant decrease in cell viability was observed in cells treated with algae extract at concentrations of 500 µg/mL and 800 µg/mL. The remaining analyzed concentrations did not cause a decrease in viability, or even resulted in a slight increase in the analyzed parameter.

Selectivity Index calculated for MCF-12A and MCF-7 cell lines treated with algae extract amounted to about 2.66.

Indole-3-acetic acid after 24 h of incubation in the MCF-7 cell line was cytotoxic at high concentrations, such as 131.39 μg/mL and 175.18 μg/mL, causing a decrease in cell viability by about 80% (Figure 2). At concentrations of 35.04 μg/mL and 87.59 μg/mL, cell viability was about 50%. Noticeable increases in viability occurred at lower concentrations, such as 0.09 μg/mL, 0.13 μg/mL, 0.18 μg/mL, and 1.78 μg/mL. In the case of treatment of the MCF-12A line with the tested plant hormone, after 24 h of incubation, an increase in the level of cell viability was noted in the concentration range of 0.09 μg/mL to 0.18 μg/mL, while 48 h of incubation with the tested compound caused only decreases in the level of the tested parameter, especially at the highest tested concentrations.

Selectivity Index calculated for MCF-12A and MCF-7 cells treated with IAA in the selected range of concentrations amounted to about 1.

24 h incubation of MDA-MB-231 cells with indole-3-acetic acid caused a statistically significant decrease in cell viability at high concentrations of the tested compound (Figure 2). At concentrations of 87.59 μg/mL, 131.39 μg/mL, and 1175.18 μg/mL, cell viability was about 60%. An IAA concentration of 17.52 μg/mL caused a decrease in the tested parameter by about 10% after 24 h of incubation. As a result of 48 h incubation, a decrease in cell viability of about 40% was noted at concentrations of 87.59 μg/mL, 131.39 μg/mL, and 175.18 μg/mL. The greatest increase in cell proliferation was observed at a concentration of IAA of 3.5 μg/mL. After 24 h of incubation with indole-3-acetic acid, an increase in the level of proliferation of ZR-75-1 cells was observed at lower concentrations. At a concentration of 0.13 μg/mL IAA, a decrease in cell viability of about 5% was noted. The greatest decrease in viability occurred at the highest concentrations—131.39 μg/mL and 175.18 μg/mL—and amounted to about 90%. In the longer incubation time, 48 h, the greatest decrease in viability was observed at concentrations of 87.59 μg/mL, 131.39 μg/mL, and 175.18 μg/mL, and it amounted to about 80%.

### 2.3. Casapase 3/7 Activity in Cancer Cells

**Figure 3:**
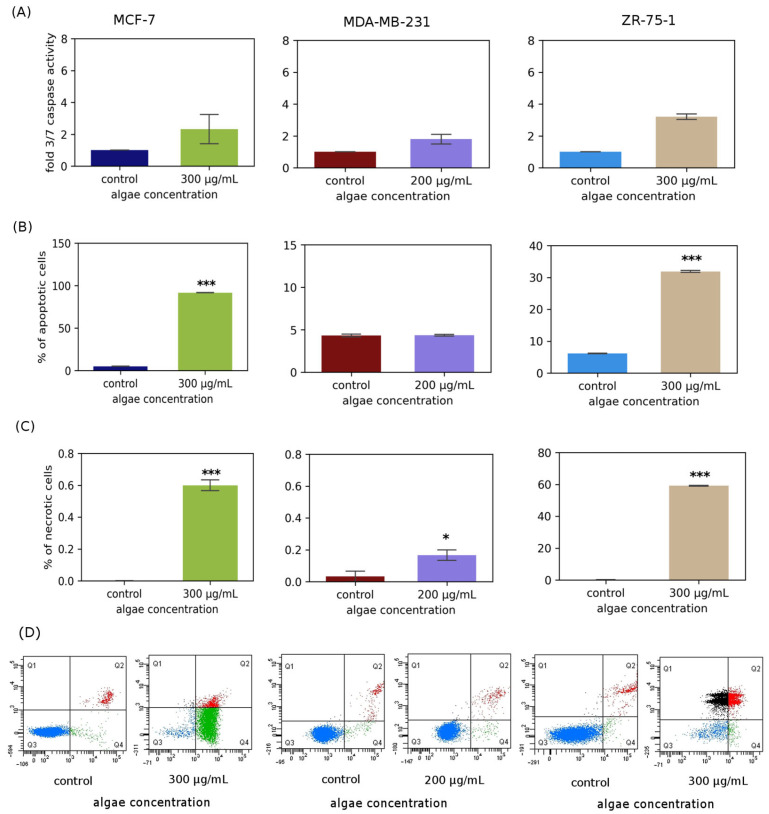
Apoptosis and necrosis studied in MCF-7, MDA-MB-231, and ZR-75-1 cell lines exposed to selected concentrations of *Chlorella vulgaris* extract for 24 h, calculated as percentage of control cells. (**A**) Luminescent assay of caspases 3/7 activity. (**B**) Percentage of apoptotic cells analyzed by flow cytometry. (**C**) Percentage of necrotic cells analyzed by flow cytometry. (**D**) Representative plots from flow cytometry analysis (Q1 black—necrotic cells, Q2 red-late apoptotic cells, Q3 blue-live cells, Q4 green-early apoptotic cells). Data are presented as mean ± standard error of mean of four replicates. *—*p* < 0.05, ***—*p* < 0.001 represent statistically significant differences from control, assessed by Dunnett’s test.

Before performing the Caspase-Glo^®^ 3/7 assay (Promega, Madison, WI, USA), cells were treated with 300 µg/mL of *Chlorella vulgaris* extract for the MCF-7 and ZR-75-1 lines and 200 µg/mL for the MDA-MB-231 line (Figure 3A). Treatment with the algal extract induced apoptosis in the MCF-7 and ZR-75-1 cell lines, significantly increasing the activity of the analyzed caspases. However, the least significant changes in caspase 3/7 activity induced by the extract were observed when the extract concentration was approximately 200 µg/mL when the extract was applied to the MDA-MB-231 cell line.

### 2.4. Flow Cytometry Analysis

Apoptosis was also assessed using flow cytometry, and the results are presented in Figure 3B. Figure 3B shows the percentage of apoptotic cells cultured for 24 h with *Chlorella vulgaris* extract at a concentration of 300 µg/mL for the MCF-7 and ZR-75-1 cell lines and at a concentration of 200 µg/mL for the MDA-MB-231 cell line. The values obtained indicate that the tested extract enhanced apoptosis to a varying extent depending on the concentration used and the cell line. Similarly to the analysis of caspase activity, flow cytometry studies also showed an increased level of apoptosis in the MCF-7 and ZR-75-1 lines, whereas a very low level of apoptotic cells was noted for the MDA-MB-231 line treated with the *Chlorella vulgaris* extract. The results obtained in the flow cytometry experiment confirmed the tested caspase activity.

### 2.5. ROS Level in Cancer Cells

**Figure 4:**
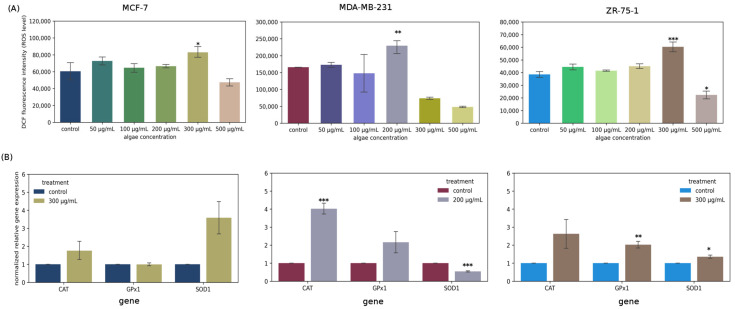
Oxidative stress parameters studied in MCF-7, MDA-MB-231, and ZR-75-1 cell lines exposed to selected concentrations of *Chlorella vulgaris* extract for 24 h. (**A**) ROS level. (**B**) Normalized gene expression. Data are presented as mean ± standard error of mean of four replicates. *—*p* < 0.05, **—*p* < 0.01, ***—*p* < 0.001 represent statistically significant differences from control, assessed by Dunnett’s test.

Figure 4A shows the effect of *Chlorella vulgaris* extract on ROS production in MCF-7, ZR-75-1, and MDA-MB-231 breast cancer cells. Incubation of cells with the tested extract caused an increase in ROS content, particularly at concentrations at which the highest cancer cell mortality was observed. At a concentration of 200 µg/mL, the extract caused an increase of approximately 38% after 24 h of exposure in the MDA-MB-231 cell line. Treatment with 300 µg/mL of the extract significantly increased the amount of ROS in MCF-7 and ZR-75-1 cell cultures compared to control cells by 37% and 56%, respectively. The presented data indicate an enhancing effect of the extract on ROS formation, which may be related to the induction of apoptosis via oxidative-stress-related pathways.

### 2.6. Oxidative Stress Enzymes Gene Expression

Figure 4B presents normalized relative gene expression of antioxidative enzymes such as catalase, glutathione peroxidase, and superoxide dismutase in the three cell lines exposed to selected concentrations of algae extract. A particularly intense increase in the expression level of the CAT-encoding gene was observed in the MDA-MB-231 cell line, which may correlate with its resistance to the extract used. Other cell lines exposed to the extract show a significantly weaker response in terms of antioxidant enzymes.

### 2.7. Cytotoxicity Tested in Bacterial and Fungal Strains

**Figure 5:**
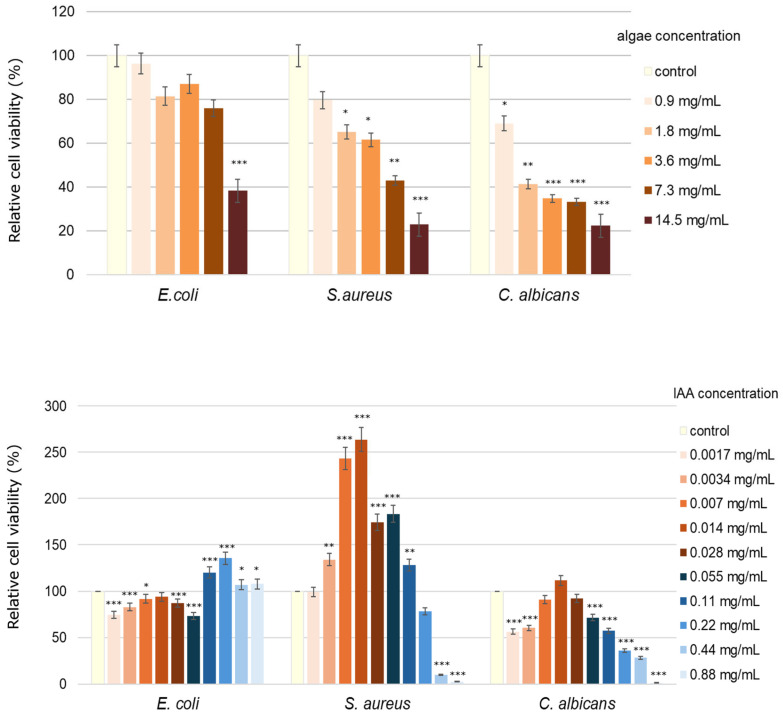
The algae extract cytotoxicity and IAA cytotoxicity studied in *E. coli*, *S. aureus*, and *C. albicans* exposed to increasing concentrations for 24 h, calculated as percentage of control. Data are presented as mean ± standard deviation of four replicates. *—*p* < 0.05, **—*p* < 0.01, ***—*p* < 0.001 represent statistically significant differences from control, assessed by Dunnett’s test.

As shown in Figure 5, IAA caused a significant inhibition of the *E. coli* growth from 6 to 27% at lower concentrations (0.0017 mg/mL, 0.0034 mg/mL, 0.07 mg/mL, 0.014 mg/mL, and 0.06 mg/mL). When analyzing the results obtained for *S. aereus*, it was observed that the inhibitory effect on bacterial growth was at concentrations between 0.22 mg/mL and 0.88 mg/mL. Regarding *C. albicans*, there was a reduction in relative cell viability, which ranged from 1% (at a concentration of 0.88 mg/mL) to 71% (at a concentration of 0.06 mg/mL) compared to the control.

Compared to the control, the relative viability of *E. coli* was reduced by 4% at a concentration of 0.9 mg/mL and by 62% at 14.5 mg/mL when the algae extract was used (Figure 5). Analysis of the results obtained for *S. aureus* revealed a decrease in viability at all concentrations of algae extract used. The lowest relative viability was observed at the two highest concentrations: 57% at 7.3 mg/mL and 77% at 14.5 mg/mL. Of the microorganisms used, *C. albicans* was the most sensitive to the algae extract. Its relative viability was below 50% at a concentration of 1.8 mg/mL compared to the control (Figure 5).

### 2.8. MIC (Minimum Inhibitory Concentration) and MBC (Minimum Bactericidal Concentration) Tested in Bacterial and Fungal Strains

As shown in Table 2, of the compounds tested, only the algae extract demonstrated activity against the strain *S. aureus* and *C albicans* (MIC = 14.5 mg/mL). The IAA exhibited antimicrobial activity against only *S. aureus* and *C albicans* (MIC = 0.44 mg/mL and MBC = 0.88 mg/mL).

### 2.9. Statistical Analysis of Obtained Results

**Figure 6:**
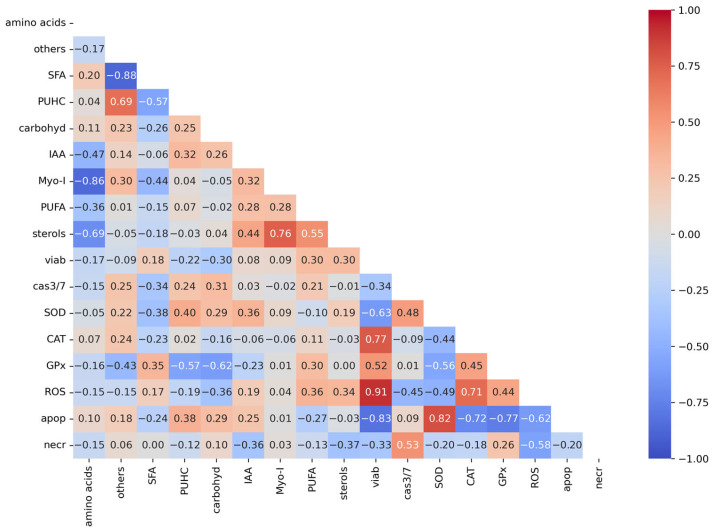
Correlation heatmap showing the relationships between the analyzed variables in breast cancer cell lines. (Explanations: SFA—saturated fatty acids, PUHC—polyunsaturated hydroxycarbons, carbohyd—carbohydrates, Myo-I—myoinositol, PUFA—polyunsaturated fatty acids, viab—cell viability, cas3/7—fold caspases 3/7 activity, apop—% of apoptotic cells, necr—% of necrotic cells, CAT—CAT gene expression, GPx—GPx gene expression, SOD—SOD gene expression).

Figure 6 shows the relationships between variables in breast cancer cell lines. Pearson correlation analysis indicates positive high correlation coefficients between ROS content and cell viability (r = 0.91), and SOD-encoding gene expression and apoptosis level (r = 0.82). Negative correlations were noted between carbohydrate content in algae extract and GPx-encoding gene expression (r = −0.62). There was also a relatively strong positive correlation between SOD gene expresion and polyunsaturated hydrocarbon content in algae extract.

## 3. Discussion

In recent years, diets based mainly on natural products of plant origin and the application of natural compounds in the prevention and treatment of cancers, as well as in the treatment of infections caused by bacterial and fungal pathogens, have enjoyed great popularity. A large number of studies conducted on the use of plant-derived substances result from the fact that effective chemotherapeutic drugs obtained by chemical synthesis have many disadvantages and cause serious side effects in the patient’s body, e.g., drug resistance caused by long-term treatment and cumulative toxicity. There are many plant sources of natural components that provide potential compounds for fighting cancers and bacterial and fungal infections. These include microorganisms, marine organisms, and plants. Of particular interest are the metabolic products of microalgae, which are increasingly being studied not only to understand their nature but also to identify substances with potential applications in various areas of human life, including health and well-being. *Chlorella* sp. is a microalga whose potential is already being exploited in the pharmaceutical, feed, dietary supplement, aquaculture, and cosmetology industries. It is relatively easy to cultivate and harvest and possesses many beneficial characteristics that enable an environmentally friendly approach to drug discovery, overcoming the challenges associated with overexploitation of marine resources [11,12,13]. Extracts from various algae species have been studied for their significant effectiveness in preventing various diseases and as adjuncts to their treatment. Research has focused primarily on the chemical composition and phytochemical, antioxidant, and pharmacological properties of algae [14]. It is important to emphasize that cancer has become one of the most significant and common diseases in the world, with significant social and economic implications. Current cancer incidence rates indicate that one in seven deaths worldwide is caused by cancer [15]. Chemotherapy, the most common treatment for cancer, has numerous side effects, including nonspecificity, resistance to treatment, and immune responses in patients, as well as numerous complications, including cardiac complications. Extracts obtained from various algae species are proving to be an extremely valuable source of biologically active substances, playing an important role in the search for new drugs. Currently, the percentage of drugs derived from natural sources that demonstrate anticancer potential is estimated at approximately 60% [16,17]. Our own studies determined the biological effect of *Chlorella vulgaris* extract on three cell lines: MCF-7, ZR-75-1, and MDA-MB-231. Cytotoxicity studies indicate that in MCF-7 and ZR-75-1 cells, the extract was most effective at a concentration of 300 μg/mL, reaching IC50, while for the MDA-MB-231 line, the IC50 was not achieved, but the extract had the strongest effect at a concentration of 200 μg/mL. Taking into account the MCF-12A cell line, it should be noted that the algae extract was cytotoxic at concentrations of 500 µg/mL and 800 µg/mL, which may mean that lower concentrations are safe to use. Selectivity Index calculated as a ratio of IC50 for healthy cells and IC50 for cancer cells treated with algae extract amounted to about 2,6, which means that the studied extract could be more toxic to cancer cells than to normal cells, indicating greater selectivity. Higher SI values indicate greater anticancer specificity, especially when the SI is higher than 3. Evaluation of SI is very crucial for determining whether further works should be performed. In our case, it is not 3, but around this value; therefore, we think that the analyzed extract is worth further studies. According to [18], extracts from *Chlorella* sp. S14 were effective at a concentration of 150 μg/mL in MCF-7 cells, which is lower than the effective concentration that we obtained. On the other hand, Sawasdee N et al. indicated that ethanolic *Chlorella* algal extract at a concentration of approximately 300 μg/mL exhibited anticancer properties, also in breast cancer cells [19]. This is in agreement with our results. Our own research also determined the effect of pure IAA on the proliferation of three cell lines: MCF-7, ZR-75-1, and MDA-MB-231. The aim was to investigate whether IAA could be a biologically active factor in the extract’s anticancer activity. Quantitative LC/MS analysis identified IAA in the tested extract at a level of 0.22 μg/mL, which means that it is most likely not the compound responsible for the extract’s anticancer activity. Studies of IAA alone confirm this finding. At the concentration found in the extract, IAA does not exhibit cytotoxic activity against MCF-7, MDA-MB-231, and ZR-75-1 cancer cells. The SI value for IAA is also very low, approximately 1, indicating low specificity and selectivity of the tested compound. Therefore, biological studies for this compound were not continued or expanded. Indole-3-acetic acid (IAA) is the most common naturally occurring auxin in plants. This commonly known, non-toxic chemical of plant origin also has a potentially cytotoxic effect on breast cancer cells. Idole-3-acetic acid itself is a non-toxic prodrug, but after activation by horseradish peroxidase, it creates a radical toxic to cancer cells, thus showing anticancer activity [20]. Our results also indicate that IAA is relatively toxic at high concentrations, while at low concentrations, it can even stimulate cell proliferation. According to the literature, a new strategy for cancer treatment is being investigated by combining indole-3-acetic acid (IAA) and horseradish peroxidase (HRP). The strength of the IAA/HRP system is that IAA itself does not cause any toxic effects on cells, while IAA produces ROS in reaction with HRP, which can effectively inhibit proliferation and induce apoptosis of cancer cells [21]. In each line tested, the intended effect of reduced cell viability was observed, but a tendency towards increased cell proliferation was also observed. In most cases, the differences were small and similar to the control. This is consistent with literature data indicating the small cytotoxic effect of IAA in various cancer cell lines, including prostate adenocarcinoma line PC-3, liver cancer line SK-HEP-1, melanoma line G361, and melanoma line B16F10 [22,23]. In all the above cases, to achieve the desired cytotoxic effect on cancer cells, IAA must be activated, e.g., by oxidative decarboxylation catalyzed by HRP. Such an activation process triggers the production of indole, skatole, and lipid peroxide free radicals. Park K.C. et al. also noticed that activation of IAA with UV light can increase its cytotoxic activity [23]. However, the mechanism of action of IAA is still not fully understood and requires further analysis. Literature data indicate a cytotoxic effect of IAA conjugated with HRP on melanoma cells of the G361 line. The mechanism of action of the analyzed compounds was associated with the induction of oxidative stress. However, it should be noted that the active factor was hydrogen peroxide, while IAA itself did not show cytotoxic effects [24]. A significant increase in the rate of cell proliferation, significantly different from the control sample, was observed in the MDA-MB-231 cell line after 48 h of incubation. This increase was also observed after 48 h of incubation of MCF-7 cells with IAA. In most cases, plant hormones and indoles have antiproliferative properties; however, it is important to note that the effects can be context-dependent, and a specific indole compound like IAA may promote invasiveness in certain cancer types [25]. It can be assumed that the level of oxidative stress can be a critical determinant in breast cancer development, as it may act pro-tumorigenic on one hand, but also pro-apoptotic on the other. Oxidative stress may cause adaptation of cancer cells, an increase in proliferation of cancer cells, and genetic instability, which leads to strong resistance to anticancer therapy [26]. The highest increase in the level of gene expression encoding antioxidant enzymes was observed in the case of the MDA-MB-231 cell line, which is most resistant to algae extract. So, we can conclude that in the case of this cell line, the tested extract is characterized by the least activity, which could be connected with MDA-MB-231’s ability to combat very high levels of oxidative stress. Most studies have emphasized the indisputable relationship between oxidative stress and the initiation, promotion, and progression of cancer [27]. Increased ROS levels are an extremely important factor in the pathogenesis of cancer, as they result in the occurrence of genetic and epigenetic modifications, which then cause uncontrolled cell proliferation [28,29,30,31]. Increased production of free radicals, which we clearly observe in our research under the influence of algae extract, leads to reduced antioxidant ability and is associated with induction of apoptosis. This is in line with literature data on changes in oxidative stress levels in breast cancer cells [32]. An increased level of ROS may induce apoptosis mainly thorough the mitochondrial pathway. Mitochondria can be damaged by high levels of ROS, which leads to the loss of mitochondrial membrane potential, and subsequently the release of cytochrome c [33]. The release of cytochrome c initiates the formation of apoptosome, which activates the executioner caspases, including caspase 7 (executioner), which participate in apoptotic cell death. This could be one of the possible mechanisms by which the *Chlorella vulgaris* extract at 300 mg/mL for the MCF-7 and ZR-75-1 cell lines and at a concentration of 200 mg/mL for the MDA-MB-231 cell line acted as an inductor of the apoptosis process. Studies on changes in caspase activity have been confirmed by analyses from flow cytometry.

Literature data indicate the induction of apoptosis by *Chlorella* sp. extract by inhibition of the act/mTOR signal pathway. Research was conducted on several different lines of cancer cells, including on the MCF-7 line [19]. The observed increase in executive 3 and 7 caspase activity correlates with the activation of both apoptotic pathways, both the extrinsic and intrinsic pathway. The reduction in cancer cell proliferation induced by the extract appears to be due to apoptosis and not necrosis, as confirmed by the results obtained using flow cytometry.

*Chlorella*, which we also showed as a result of chemical analyses (Table 1) is a rich source of many bioactive compounds, which, when acting synergistically, show anticancer effects. Kubatka P. et al. confirmed the anticancer effect of the *Chlorella pyrenoidosa* species in their research on rats [34]. We identified numerous bioactive chemical compounds in the extract, including numerous amino acids, carbohydrates, unsaturated fatty acids, sterols, and many others. These compounds have proven biological activity, and their presence in the tested extract correlates with the effects observed in cancer cells [35]. Rzeska et al. also observed anticancer properties of *Chlorella* extract based on the inhibition of endometrial cancer cell viability [36]. Our statistical analysis of the results indicates a positive correlation between the level of saturated fatty acids present in the extract and the activity of antioxidant enzymes, primarily GPx. We also observed a positive correlation between increased ROS levels in cancer cells and the presence of PUFAs and plant sterols in the extract. This confirms the well-known hypothesis that unsaturated fatty acids, similarly to sterols, possess anticancer properties. PUHC compounds also appear to enhance apoptosis in cancer cells, while hydrocarbon compounds reduce the viability of breast cancer cells.

The apparent link between the anticancer and antimicrobial properties of algae extracts should be emphasized, which may be due to the fact that both effects are often mediated by the same classes of potent bioactive secondary metabolites produced by algae. Algae have evolved to produce a variety of chemical compounds as a defense mechanism against pathogens, competitors, and environmental stressors, which may ultimately lead to therapeutic potential in the treatment of both infections and cancer. An example of such compounds could be polyphenols, which induce apoptosis in cancer cells and disrupt microbial cell membranes and inhibit essential microbial enzymes [37,38]. The increasing resistance of microorganisms to antibiotics is a growing problem that concerns people worldwide. This situation can pose a serious health threat, particularly in rapidly developing countries where antibiotics are prescribed in large quantities [39]. Therefore, using algae extracts seems like a good solution. They could be a future alternative to antibiotics, as they are an excellent source of various biological compounds with a pool of unique secondary metabolites [40]. Our own research indicates a significant effect of algae extract on the growth of microorganisms. As reported by Yuvaraj et al. [41], the use of *Cladophora* species extract resulted in inhibition of the growth of various groups of microorganisms such as *Enterococcus* sp., *Streptococcus agalactiae*, *Vibrio fischeri*, *Vibrio parahaemolyticus*, *Vibrio anguillarum*, and *Vibrio vulnificus*, depending on the season in which the algae samples were collected. In turn, Stabili et al. [42] noticed that the extract of *C. rivularis* (Linneaus) Kuntze has the potential to inhibit the growth of Gram-negative and Gram-positive bacteria, including *Staphylococcus aureus*. Krish and Das [43], after applying *Cladophora rupestris*, observed inhibition of the growth of microorganisms such as *Pseudomonas aeruginosa*, *E. coli*, *Vibrio parahaemolyticus*, *V. harveyii*, and *V. alginolyticus*. A similar relationship was observed in our own studies, where the applied *Chlorella vulgaris* extract, at a concentration of 0.9 mg/mL, inhibited the growth of *S. aureus* by 20% compared to the control. In *E. coli*, however, a concentration of 1.8 mg/mL began to inhibit bacterial growth by approximately 20%, and at the highest applied concentration, a significant decrease in the relative viability of *E. coli* was observed by approximately 60% compared to the control. The inhibition of the growth of the tested bacteria could be caused by the presence of linolenic acid in the algae extract, which could influence the inhibition of the growth of *S. aureus* and *E. coli*, which was confirmed by the study of Stabili et al. [42]. Literature data indicate that *C. vulgaris* extracts, due to their composition, also have antifungal properties. [44,45]. The results of this study prove that the extract of *C. vulgaris* is effective against *C. albicans* as each concentration of the extract used inhibited its growth, which was also confirmed by Ahmed 2016 [45]. In our extract of *C. vulgaris*, the content of various bioactive compounds, especially fatty acids, could be responsible for the morphological changes in *C. albicans* and contribute to membrane damage and its degradation. Jyotirmayee et al., 2014 [46] states that fatty acids with C-10 and higher can contribute to protoplast lysis and the breakdown of cell membranes, thus causing leakage of cytoplasm with cell contents, leading to cell death. Furthermore, as noted by Stramarkou et al., 2017, not all extracts obtained from *C. vulgaris* will have a similar effect on microorganism structures, as the bioactivity of the extract significantly depends on the solvent used, and the results obtained in different studies may differ [47].

Our research results are consistent with the literature data indicating the antibacterial effect of IAA. The antibacterial activity of IAA has also been demonstrated against *Pseudomonas aeruginosa*. Jayaraman et al. showed that IAA is an effective antibiofilm agent against *P. aeruginosa* [48]. It is worth mentioning that IAA exhibited significant bactericidal activity against Gram-positive bacteria (*Staphylococcus aureus*) and Gram-negative bacteria (*Escherichia coli*) [49]. Our studies also confirmed the antifungal activity of IAA, which is consistent with the literature data. Chitra et al. demonstrated such activity of IAA against *Aspergillus fumigates*, *Rhizopus oryzae*, and *Candida albicans* [50]. The application of IAA at various concentrations maintained the relative viability of *E. coli* at a level similar to control cells. As indicated by Toner et al., Gram-negative bacteria have the ability to produce enzymes and other metabolites that allow them to use phytohormones as an energy source [51]. IAA can affect Gram-negative bacteria by influencing changes in their metabolism and gene expression, which leads to the resistance of such bacteria to high concentrations of the phytohormone in the environment [51]. This is confirmed by our own studies, where *E. coli* did not show sensitivity to the effects of the phytohormone IAA. In the case of *S. aureus* bacteria, the applied concentrations of IAA significantly stimulated the growth and viability of bacteria compared to the control. Studies conducted by Cunha et al. [52] indicate that auxins may exhibit some antimicrobial properties against *S. aureus*, which was observed only above a concentration of 1.25 mM in our own studies. In turn, Rico-Jiménez et al. [53] indicate that high-throughput transcriptomic studies showed that genes related to amino acid, nucleotide, carbohydrate, and lipid metabolism were most susceptible to IAA action, which translated into energy and amino acid metabolism in antibiotic-resistant *Staphylococcus aureus*. These studies suggest that indole-3-acetic acid may be a potential antimicrobial agent against Gram-positive bacteria such as *Staphylococcus aureus*, especially at higher concentrations, by disrupting cellular metabolic processes. Literature data indicate that some phytohormones exhibit toxic effects on *Candida albicans* [54]. This is due to the fact that the *C. albicans* cell wall is a dynamic structure that changes in response to environmental conditions. The regulation of cell wall biosynthesis and remodeling is controlled by many signaling pathways and transcriptional regulators, such as Sfp1, which controls the expression of genes related to the response to cell wall stress [55]. The above processes may explain the inhibitory effect of IAA on *C. albicans* when low and high concentrations of IAA (0.01 mM and 0.02 mM and from 0.31 to 5.0 mM) are used. The results obtained from MIC and MBC tests confirm those from cytotoxicity analysis, indicating relatively high concentrations of both algae extract and IAA needed for antimicrobial activity.

Despite the very interesting results we obtained, it is important to point out certain limitations of the presented experiment. It is worth noting that the in vitro model represented by the tested cell lines is a 2D model, lacking the tumor microenvironment, which includes extracellular matrix components and various chemical components, and which significantly alters the metabolism of cancer cells. Furthermore, cells in culture exhibit certain changes in phenotype, metabolism, and signaling pathways compared to cells in the tumor. This also represents a significant pharmacokinetic and pharmacodynamic simplification. It is also worth emphasizing that algae extracts often have unstable compositions resulting from environmental factors, culture methods, extract production methods, and even storage. Furthermore, the extract is a mixture of numerous, often unidentified, compounds, and we do not fully understand which of them causes the observed effect.

However, the presented results encourage further clinical studies as well as detailed chemical analyses to determine the phytochemical composition of the extract.

## 4. Materials and Methods

### 4.1. Reagents

DMEM (Dulbecco’s Modified Eagle Medium) culture medium, Leibovitz L-15 culture medium, RPMI—1640 culture medium, 0.25% trypsin 1 mM EDTA, fetal bovine serum (FBS) (Gibco, San Diego, CA, USA), penicillin/streptomycin antibiotic mixture—10,000 units/mL penicillin, 10,000 µg/mL streptomycin, and MTT (Sigma-Aldrich, St. Louis, MO, USA) were used in this study.

### 4.2. Biological Material

The study used algae of the species *Chlorella vulgaris* BA02 from the Culture Collection of Baltic Algae (CCBA) from the Institute of Oceanography, Faculty of Oceanography and Geography, University of Gdańsk in Gdynia.

### 4.3. Research Site and Algae Cultivation Method

The algae cultivation method was based on Makowska (2017), Huang et al. (2016), and Silva et al. (2023) [56,57,58]. The ATCC (American Type Culture Collection) Medium 616 culture medium—BG 11 (Blue Green Algae) substrate was used to cultivate *Chlorella vulgaris* BA02 algae. The medium consists of the following chemicals per liter of distilled water: 1.5 g NaNO_3_, 0.04 g K_2_HPO_4_, 0.075 g MgSO_4_ × 7H_2_O, 0.036 g CaCl_2_ × 2H_2_O, 6.0 mg Citric acid, 6.0 mg Ferric ammonium citrate, 1.0 mg EDTA, 0.02 g Na_2_CO_3_, 1.0 mL Trace Metal Mix A5 (which consists of 2.86 g H_3_BO_3_, 1.81 g MnCl_2_ × 4H_2_O, 0.222 g ZnSO_4_ × 7H_2_O, 0.039 g Na_2_MoO_4_ × 2H_2_O, 0.079 g CuSO_4_ × 5H_2_O, 49.4 mg Co(NO_3_)_2_ × 6H_2_O, per 1000.0 mL of distilled water) (source: https://www.atcc.org/, accessed on 11 April 2025). Algae cultivation was carried out in a New Brunswick BioFlo 310 bioreactor (New Brunswick Scientific, Eppendorf AG, Edison, NJ, USA) in a periodic type (one-time preparation of the culture medium). The volume of the culture medium for algae multiplication was 5 L. The bioreactor was illuminated with two warm white (3000 K) LED (light-emitting diode) panels with a power of 18.5 W (watts) per panel. The LED panels were set at a distance from the bioreactor so that the intensity of photosynthetic radiation (PAR) was 500 µmol∙m^−2^∙s^−1^, which is 200 µmol∙m^−2^∙s^−1^∙L^−1^ of the culture medium. The light was on for 10 h and off for 14 h. The algal growth period was 3 weeks, after which the algal biomass was collected for preparing extracts.

### 4.4. Extraction Method from Microalgae

The collected algal biomass was concentrated by gravity sedimentation and centrifugation at 2000 RPM for 10 min. Then, the concentrated biomass was dried in a dryer at 30 °C. The dry algal biomass was torn in an agate mortar, weighed, and transferred to a 50 mL Falcon tube in which the extraction was carried out.

A modified ultrasonic lipid extraction method described in Figueiredo et al. (2019) was used to extract IAA from microalgae *Chlorella vulgaris* BA02 [59]. The extraction was carried out in methanol. The Sonics Vibra Cell ultrasonic probe was used for extraction. The extraction process was carried out at the following settings: amplitude 95%, 30 s of pulses, and 20 s of rest. The total sonication time was 6 min. The tube with algal biomass was placed in a cooler rack throughout the sonication period to cool it down. Then, the tube was centrifuged at 2000 RPM for 10 min. The supernatant was poured into a new tube, and pure methanol was added to the pellet and the sonication process was repeated. These steps were repeated 10 times until the extraction solvent lost its green color. The obtained supernatants were combined into one mixture and the extraction solvent was evaporated on an evaporator at 36 °C and 260 bar [59].

### 4.5. Chemical Analysis of Chlorella Vulgaris Extract—GC/MS Qualitative Analysis

5 mg of the dry residue after extraction was redissolved in 220 µL of anhydrous pyridine and 80 µL of BSTFA (Ν,O-bis-(trimethylsilyl)-trifluoroacetamide), and then the mixture was heated at 80 °C for 50 min. Solutions were analyzed by GC-MS (Agilent GC System 7890B coupled to an Agilent MS Triple Quad 7000C; Agilent Technologies, Santa Clara, CA, USA). Chromatographic separation was performed on an HP-5MS (30 m × 0.25 mm × 0.25 µm) fused silica capillary column at a helium constant flow rate of 1 mL/min. The injector worked in split mode (1:10), and the injector temperature was 260 °C. The initial column oven temperature was 50 °C, and increased to 320 °C at a rate of 3 °C/min, and the final temperature was held for 10 min. The ion source temperature was 230 °C and the quadrupole temperature was 150 °C. The ionization energy (EI) was 70 eV. Detection was performed in full-scan mode over a range of 41–650 m/z. Identification was carried out on the National Institute of Standards and Technology (NIST) basis and the calculated retention index. After integration, the percentage of each component in the total ion current (TIC) was calculated [60].

### 4.6. IAA Estimation in Algae Extract with the Use of LC/MS

10 mg of the dry residue was redissolved with 1 mL of mobile phase, centrifuged at 4500 rpm for 10 min, and filtered through a 0.45 μm filter (Whatman GF/C; Sigma-Aldrich, St. Louis, MO, USA). The solution was analyzed by ESI-LC-MS/MS (6420 LC/MS Triple Quadrupole, Agilent Technologies, Santa Clara, CA, USA) according to [61].

### 4.7. In Vitro Cell Culture Methodology and Main Principles of the Experiment

Three studied breast cancer cell lines (MCF-7, MDA-MB-231, and ZR-75-1) and one healthy breast cell line, MCF-12A, were obtained from American Type Culture Collection (ATCC, Manassas, VA, USA). The cells were cultured in complete growth medium supplemented with heat-inactivated fetal bovine serum (FBS)–10% *v*/*v*, with the addition of antibiotics: streptomycin (10,000 U/mL) and penicillin (10 mg/mL), under standard conditions in a CO_2_ incubator (temperature 37 °C, 5% CO_2_, 100% relative humidity). The adherent MCF-7 tumor line was cultured in DMEM culture medium supplemented with FBS (10%, *v*/*v*). The ZR-75-1 line was cultured in RPMI-1640 culture medium supplemented with FBS (10% *v*/*v*), while the MDA-MB-231 line was cultured in Leibovitz L-15 culture medium supplemented with FBS (10%, *v*/*v*) without CO_2_. MCF-12A cells were cultured in DMEM/F12 medium that was supplemented with 5% horse serum, 0.1 µg/mL cholera toxin, 40 ng/mL epidermal growth factor, and penicillin (100 U/mL) and streptomycin (100 µg/mL) at 37 °C in a humidified atmosphere of 5% CO_2_ in air. Cells of all lines were maintained in the logarithmic growth phase by regular passage to new culture vessels when the cultures reached approximately 80% confluence. Cells were seeded onto sterile 96-well culture plates. Cells were cultured 24 h before the planned experiment to allow them to adhere to the culture vessel surface and multiply.

### 4.8. Evaluation of Cytotoxicity of the Algae Extract and IAA

In order to determine the cytotoxicity of algae extract and IAA towards the tested cell lines, the MTT test was used.

Cells were seeded onto sterile 96-well microplates, and after 24 h of culturing, algae extract and IAA were added to the designated microplate wells and incubated for 24 h (for both algae extract and IAA) and 48 h for IAA. Cells of the three studied cell lines were treated with algae extract in 6 concentrations: 50 μg/mL, 100 μg/mL, 200 μg/mL, 300 μg/mL, 500 μg/mL, and 800 μg/mL, and with IAA in 11 concentrations: 0.09 μg/mL, 0.13 μg/mL, 0.18 μg/mL, 1.78 μg/mL, 3.5 μg/mL, 8.76 μg/mL, 17.52 μg/mL, 35.04 μg/mL, 87.59 μg/mL, 131.39 μg/mL, and 175.18 μg/mL. IAA concentration during the experiment in the extract was at 0.22 μg/mL. The concentration of *Chlorella vulgaris* extract for the study was selected based on literature data on microalgae extracts and their anticancer activity. The data indicate a very wide range of concentrations of the tested extracts, reaching up to 2000 μg/mL [62,63,64]. The medium was then removed, and the cell monolayer was washed twice with PBS. MTT tetrazolium salt solution at a final concentration of 0.05 mg/mL was added to each microplate well. After incubation, DMSO was added to the microplate wells to dissolve the purple formazan crystals formed in the living cell environment. The absorbance of the formazan solution, after gentle mixing, was read at a wavelength of λ = 560 nm on a Glomax Promega microplate reader. Live cells were calculated by comparing the absorbance value of the tested samples with the absorbance value of the control (cells not treated). The absorbance of the control was taken as 100%. To calculate IC50, we used a dose–response curve and performed a manual interpolation on a graph. All the experiments were performed in triplicate. In order to assess the safety and efficacy of the studied extracts and IAA, Selectivity Index (SI) was calculated as the ratio of the IC50 of normal cells (MCF-12A) to the IC50 for cancer cells (MCF-7). Based on the obtained results, only algae extract was chosen for further studies.

Algae extract concentrations for further experiments were chosen based on the results obtained in the cytotoxicity assay. The results obtained for IAA were so unsatisfactory that we decided not to continue investigating the mechanisms of action of this hormone in both human and bacterial cells. The cytotoxic effect was achieved with this compound at extremely high concentrations, making them unusable.

### 4.9. Caspase 3/7 Activity Assay

The activity of caspases 3/7 was examined at algae extract concentrations of 300 µg/mL (MCF-7 and ZR-75-1) and 200 µg/mL (MDA-MB-231) after 24 h of incubation. Breast cancer cells were seeded in 96-well white plates at a density of 2 × 10^4^ cells/well. Luminescent assay was applied according to the manufacturer’s instructions (Promega Corporation, Madison, WI, USA) as described previously [65]. A microplate reader (GloMax^®^-Multi Microplate Multimode Reader; Promega Corporation, Madison, WI, USA) was used and the experiments were performed in triplicate.

### 4.10. Analysis of Apoptosis Using Flow Cytometry

The apoptosis of the MCF-7, MDA-MB-231, and ZR-75-1 cell lines exposed to algae extract (200 µg/mL for MDA-MB-231 and 300 µg/mL for MCF-7 and ZR-75-1) were evaluated by flow cytometry on the FACSCanto II cytometer (BD, San Diego, CA, USA) according to [65]. The MCF-7, MDA-MB-231, and ZR-75-1 cells (2.0 × 10^5^ per well) were seeded in 2 mL of medium in six-well plates. After 24 h, the medium was removed, and replaced with algae extract concentrations of 300 µg/mL for MCF-7 and ZR-75-1 cells, and 200 µg/mL for MDA-MB-231 cells in the medium. The cells were incubated for 24 h. The cells were detached, resuspended in the medium, and then resuspended in a binding buffer. Subsequently, the cells were stained with FITC Annexin V and PI (FITC Annexin V apoptosis detection Kit I (BD Pharmingen^TM^, San Diego, CA, USA)) at room temperature in the dark for 15 min. The data were analyzed using FACSDiva software version 6.1.3 (BD Pharmingen^TM^, San Diego, CA, USA). All the experiments were performed in triplicate.

### 4.11. Selected Gene Expression Evaluation with the Use of RT-qPCR

In order to analyze GPx (glutathione peroxidase), SOD (superoxide dismutase), and CAT (catalase) relative gene expression in MCF-7, MDA-MB-231, and ZR-75-1 cells, RT-qPCR was applied [66]. Cells were exposed to different concentrations of algae extract: 200 µg/mL for MDA-MB-231 and 300 µg/mL for MCF-7 and ZR-75-1; RNeasy Mini Kit (QIAGEN, Hilden, Germany) was used with the QIAcube System (QIAGEN, Hilden, Germany) in order to obtain the total amount of RNA from the cells. GoTaq^®^ 1-Step RT-qPCR System (Promega Corporation, Madison, WI, USA) was applied according to manufacturer guidelines. Primers for genes encoding glutathione peroxidase (GPx), superoxide dismutase (SOD1), and catalase (CAT) in human cells were purchased from the Bio-Rad collection as PrimePCR™ SYBR^®^ Green Assay (Bio-Rad Laboratories GmbH, Munich, Germany). GAPDH was used as a reference gene, and its sequence and properties have been published by Piana et al. [67]. Transcript levels were calculated with the use of the method by Livak and are expressed as the relative normalized expression (2^−ΔΔCt^) [68]. All the experiments were performed in triplicate.

### 4.12. Intracellular ROS Detection

The level of intracellular reactive oxygen species (ROS) was determined using dichlorodihydrofluorescein diacetate (DCFH-DA) (Sigma, St. Louis, MO, USA) [69]. Intracellular ROS levels were examined at algae extract concentrations of 200 µg/mL for MDA-MB-231 and 300 µg/mL for MCF-7 and ZR-75-1 after 24 h of incubation. Breast cancer cells were seeded in 96-well white plates at a density of 2 × 10^4^ cells/well. Dichlorodihydrofluorescein diacetate (DCFH-DA) (Sigma, St. Louis, MO, USA) and GloMax^®^-Multi Detection System (Promega Corporation, Madison, WI, USA) were used in order to measure the level of intracellular reactive oxygen species (ROS). All the experiments were performed in triplicate.

### 4.13. Evaluation of Cytotoxicity of Algae Extract and IAA in Bacterial/Fungal Cells

To determine the antimicrobial activity of the algae extract and indole-3-acetic acid (IAA), the MTT method was used, which determines the number of viable microbial cells. The studies used overnight cultures of *E. coli* (ATCC 25922), *S. aureus* (ATCC 6538), and *C. albicans* (ATCC 10231) strains at a concentration of 10^6^ cfu/mL (*E. coli* and *S. aureus*) and 10^4^ cfu/mL (*C. albicans*). Algae extract concentrations used for analysis were 0 mg/mL (control), 0.9 mg/mL, 1.8 mg/mL, 3.6 mg/mL, 7.3 mg/mL, and 14.5 mg/mL, and IAA concentrations used for analyses were 0 mg/mL (control), 0.0017 mg/mL, 0.0034 mg/mL, 0.007 mg/mL, 0.014 mg/mL, 0.027 mg/mL, 0.055 mg/mL, 0.11 mg/mL, 0.22 mg/mL, 0.44 mg/mL, and 0.88 mg/mL. Analyses were performed according to the protocol of Jabłońska-Trypuć et al. [70]. The results obtained are expressed as a percentage of relative viability of cells compared to the control. All the experiments were performed in triplicate.

### 4.14. Determination of MIC (Minimum Inhibitory Concentration) and MBC (Minimum Bactericidal Concentration)

Tests were applied according to the EUCAST (European Committee on Antimicrobial Susceptibility Testing) reference method (EUCAST. European Committee on Antimicrobial Susceptibility Testing. V. 9.0). MIC and MBC were indicated, which allowed for a reliable assessment of the bactericidal and fungicidal potential of the tested concentrations of algae extract and IAA. The MIC was determined using microdilutions in culture broth (MHB for bacteria and Sabouraud broth for yeast). The MBC was determined by transferring the microorganism from a broth culture after 24 h incubation into a solid culture medium, in order to identify the lowest concentrations of algae extract and IAA that would completely stop growth (<99%) [71]. In our studies, negative controls were used as a non-treatment microbial inoculum, while positive controls were antibiotics such as Ampicillin (10 µg/mL) for bacteria and Itraconazole (10 µg/mL) for yeast.

### 4.15. Statistical Analysis

Each determination was performed in triplicate in four independent experiments, and all results are given as mean values ± standard deviation. Differences between means were calculated using one-way ANOVA. For statistically significant differences, Dunnett’s test was performed, comparing the mean results obtained for the control and the remaining variables. Significant effects are presented as *—*p* < 0.05, **—*p* < 0.01, and ***—*p* < 0.001. Statistica 13.4 package was used to perform statistical analyses. Relationships between the studied variables for the breast cancer cell lines were examined using Pearson correlation analyses as a correlation heatmap. Analyses were prepared on Python 3.7 and Jupiter notebook as a platform.

## 5. Conclusions

The aim of the presented study was to assess the chemical composition and anticancer and antimicrobial properties of *Chlorella vulgaris* extract, and also to investigate the potential cytotoxicity of one of its components—indoleacetic acid (IAA). The study revealed that one of the main groups of chemical compounds in the extract were fatty acids known for their health-promoting properties. The extract was found to have antibacterial activity against *S. aureus* and *C. albicans*, but to a lesser extent against *E. coli*. The three breast cancer cell lines and one healthy cell line derived from breast tissue under investigation showed that the extract has anticancer properties and a relatively high SI value. The studied extract was not toxic in lower concentrations for normal healthy cells. The cytotoxic effect of the extract was found to be related to apoptosis and oxidative stress. This result was confirmed by flow cytometry analysis. The extract induced oxidative stress as evidenced by increased levels of ROS and increased expression of gene-encoding antioxidant enzymes. The increased level of SOD in MDA-MB-231 cells correlates with a high level of ROS, which explains the cell’s inability to neutralize ROS. MDA-MB-231 cells’ level of proliferation was high due to stimulation by ROS presence. This explains the highest level of MDA-MB-231 cell resistance to extraction activity. Unfortunately, our research has shown that IAA is not the compound primarily responsible for the anticancer activity of *Chlorella vulgaris* extract, as this compound alone does not exhibit the expected biological activity. However, the chemical composition of the extract indicates a multitude of possibilities in terms of biologically active compounds. Considering the results obtained, it is particularly important to emphasize the need to continue research in this direction and to extend it to investigate the molecular mechanisms by which *Chlorella vulgaris* extract exhibits such interesting and important effects. This applies to both cancer cells and microbiological studies. In an era of alarmingly rapid antibiotic resistance, we should look for alternatives to traditional antibiotic therapy, which is increasingly proving to be ineffective.

In summary, these results suggest that the extract may exert its cytotoxic effects on cancer cells by inducing oxidative stress, disrupting the balance of antioxidant enzymes, and stimulating apoptosis. However, certain limitations of the presented studies should be noted. Future studies should certainly be expanded to include isolation, purification, identification, and understanding of the mode of action of the extract’s components before they can be considered for the production of drugs and/or supplements.

## Figures and Tables

**Table 1:** Main compounds present in *Chlorella* extract estimated by using GC-MS analysis.

Compound	RT	Relative Abundances (% of TIC)	Group of Compounds
**Myo-Inositol**	50.78	0.073	
3-Indoleacetic acid (IAA) *	**44.29**	**0.004**	
**Alanine**	11.58	1.762	amino acids
**L-Proline**	14.26	0.109	
**L-Valine**	16.56	0.516	
**L-Leucine**	19.16	0.199	
**L-Proline**	19.95	0.947	
**L-Isoleucine**	20.09	0.279	
**Serine**	23.39	0.085	
**L-Threonine**	24.56	0.169	
**L-Aspartic acid**	27.97	0.123	
**Pyroglutamic acid**	29.65	0.030	
**4-Aminobutanoic acid**	30.09	1.234	
**Phenylalanine**	33.81	0.018	
**L-Glutamic acid**	34.04	0.574	
**2-Aminoheptanedioic acid**	39.56	0.353	
**Butanedioic acid**	20.91	0.960	saturated fatty acids
**Palmitic Acid**	48.37	7.533	
**Stearic acid**	54.52	0.043	
**β-Fructofuranose**	41.55	0.300	carbohydrates
**α-Psicofuranose**	41.71	0.188	
**β-Fructofuranose**	41.83	2.050	
**α-Glucopyranose**	44.49	0.956	
**β-Glucopyranose**	47.77	1.160	
**Glyceryl-glycoside**	58.06	7.713	
**D-(+)-Turanose**	65.60	0.199	
**Sucrose**	66.90	17.603	
**Neophytadiene**	41.27	0.904	polyunsaturated hydrocarbons
**Phytol**	52.47	0.713	
**Squalene**	69.62	0.415	
**4,7,10,13-Hexadecatetraenoic acid**	46.89	7.448	unsaturated fatty acids
**7,10-Hexadecadienoic acid**	47.22	0.581	
**Palmitoleic acid (Z) + 4,7,10-Hexadecatrienoic acid**	47.42	2.281	
**Palmitelaidic acid (E)**	48.10	0.761	
**Linolenic acid, methyl ester**	49.89	0.229	
**Pinolenic acid**	52.79	0.139	
**6.9,12,15-Octadecaetraenoic acid**	52.99	0.424	
**Linoleic acid**	53.39	3.513	
**α** **-Linolenic acid + Oleic Acid (Z),**	53.59	17.269	
**Oleic Acid (E)**	53.75	0.223	
**Unknow glycoside**	78.59	2.960	sterols and derivatives
**Unknow glycoside + 3-Hydroxyergost-7-ene**	80.20	1.613	
**α-Spinasterol**	80.93	0.852	
**3-Pyridinol**	12.52	0.146	others
**Ethanolamine**	18.73	0.606	
**Phosphoric acid**	19.39	0.187	
**Glycerol**	19.61	13.413	
**α-Glycerophosphoric acid**	39.93	0.144	

* 3-Indoleacetic acid (IAA)—tested plant hormone.

**Table 2:** The MIC and MBC values for tested compounds (algae extract and IAA) against selected microorganisms.

Microorganism	MIC (mg/mL)		MBC (mg/mL)		Positive Control
	Algae Extract	IAA	Algae Extract	IAA	
***Escherichia coli***	>14.5	>0.88	>14.5	>0.88	Ampicillin 10 µg/mL
***Staphylococcus aureus***	14.5	0.44	>14.5	0.88	
***Candida albicans* (yeast) **	14.5	0.44	>14.5	0.88	Itraconazole 10 µg/mL

## Data Availability

The data presented in this study are available on reasonable request from the corresponding author.
